# Molecular Analysis of *Pfs47*-Mediated *Plasmodium* Evasion of Mosquito Immunity

**DOI:** 10.1371/journal.pone.0168279

**Published:** 2016-12-19

**Authors:** Gaspar E. Canepa, Alvaro Molina-Cruz, Carolina Barillas-Mury

**Affiliations:** Laboratory of Malaria and Vector Research, National Institute of Allergy and Infectious Diseases, National Institutes of Health, Rockville, Maryland, United States of America; Kansas State University, UNITED STATES

## Abstract

Malaria is a life-threatening disease caused by *Plasmodium falciparum* parasites that is transmitted through the bites of infected anopheline mosquitoes. *P*. *falciparum* dispersal from Africa, as a result of human migration, required adaptation of the parasite to several different indigenous anopheline species. The mosquito immune system can greatly limit infection and *P*. *falciparum* evolved a strategy to evade these responses that is mediated by the *Pfs47* gene. *Pfs47* is a polymorphic gene with signatures of diversifying selection and a strong geographic genetic structure at a continental level. Here, we investigated the role of single four amino acid differences between the *Pfs47* gene from African (GB4 and NF54) and a New World (7G8) strains that differ drastically in their ability to evade the immune system of *A*. *gambiae* L35 refractory mosquitoes. Wild type NF54 and GB4 parasites can survive in this mosquito strain, while 7G8 parasites are eliminated. Our studies indicate that replacement in any of these four single amino acids in *Pfs47* from the NF54 strain by those present in 7G8, completely disrupts the ability of NF54 parasites to hide from the mosquito immune system. One of these amino acid replacements had the opposite effect on *A*. *albimanus* mosquitoes, and enhanced infection. We conclude that malaria transmission involves a complex interplay between the genetic background of the parasite and the mosquito and that *Pfs47* can be critical in this interaction as it mediates *Plasmodium* immune evasion through molecular interactions that need to be precise in some parasite/vector combinations.

## Introduction

*Plasmodium falciparum* causes the most virulent form of malaria in humans and is transmitted by anopheline mosquitoes. In 2015, there were 214 million new cases and an estimated 438,000 malaria deaths worldwide [1]. *P*. *falciparum* malaria appears to have originated in Africa from a single horizontal transfer from gorillas to humans [2] and became a global disease as infected humans migrated out of Africa. This remarkable journey led to the adaptation of the parasite to more than 70 different anopheline vector species [3]. In some cases, parasites encountered mosquito species that were evolutionarily distant from African vectors. For example, anophelines of the subgenus *Nyssorhynchus* (malaria vectors in Central and South America, such as *Anopheles albimanus*) diverged from the subgenus *Cellia* (malaria vectors in Africa, India, and South Asia) about 100 million years ago (MYA) [4].

*P*. *falciparum* isolates from different geographic origins can exhibit dramatic differences in infectivity to the same mosquito vector, suggesting that natural selection took place as parasites adapted to different mosquito vector species. Some *P*. *falciparum* lines of African origin (GB4 and NF54) can infect an *Anopheles gambiae* L35 strain (R strain) that was selected to be highly refractory to infection with *Plasmodium cynomolgi* (monkey malaria), while a line from Brazil (7G8) is eliminated and encapsulated [5, 6]. Conversely, *Anopheles albimanus* (a New World vector) is more susceptible to infection with 7G8 (Brazilian) than with African *P*. *falciparum* lines [7]. Recent studies indicate that the mosquito complement-like system is a major determinant of vector-parasite compatibility. The thioester containing protein-1 (TEP1) is a key component of the *A*. *gambiae* complement-like system that binds to the surface of *Plasmodium* ookinetes, as they come in contact with the mosquito hemolymph, activating the formation of a complex that kills the parasite [8]. In *A*. *gambiae* R strain, damaged parasites are covered with melanin [5], an insoluble black pigment, through a TEP1-dependent mechanism, while in other mosquito strains parasites can be lyzed by the complement system. TEP1 is stabilized in the mosquito hemolymph by forming a complex with two leucine-rich repeat proteins, LRIM1 and APL1C [9, 10]. Silencing either LRIM1 or APL1C results in premature, systemic activation and deposition of TEP1 or the active form (TEP1-cut) is depleted in the hemolymph [9, 10]. Disruption of the mosquito complement system in *A*. *gambiae* R strain mosquitoes, by silencing either TEP1 or LRIM1, reverts the refractory phenotype and allows *P*. *falciparum* 7G8 parasites to survive, while it has no significant effect on the prevalence or intensity of infection with NF54 and GB4 parasites [6]. This indicates that 7G8 parasites are detected and eliminated by the mosquito immune system, while most NF54 and GB4 parasites are able to evade the system and survive.

A combination of genetic linkage mapping in a cross between *P*. *falciparum* GB4 (an African strain that survives in *A*. *gambiae* R strain) and a *P*. *falciparum* 7G8 (Brazilian strain that is melanized), followed by linkage group selection and functional genomics analysis was used to identify *Pfs47* as the gene that allows African (GB4) parasites to become “invisible” to the mosquito immune system [11]. Genetic disruption of *Pfs47* in *P*. *falciparum* NF54 resulted in almost complete elimination of the parasite in *A*. *gambiae* that could be reverted by silencing TEP1 expression [11]. Global analysis of 364 different *P*. *falciparum* isolates identified 42 different *Pfs47* haplotypes with strong geographic structure. Laboratory infections showed that *P*. *falciparum* isolates from Africa, Asia, or the Americas have low compatibility to malaria vectors from a different continent that can be overcomed by disrupting the mosquito immune system [7]. Replacement of the *Pfs47* gene in NF54 (African) parasites with a *Pfs47* haplotype common in Asia or the New World made the African parasite compatible with an Asian (*A*. *dirus*) or American (*A*. *albimanus*) vector respectively, by making the parasite “undetectable” by the mosquito immune system in these vectors [7]. This indicates that *Pfs47* plays a key role in *Plasmodium*-mosquito compatibility and malaria transmission worldwide. Remarkably, there are only four amino acid differences between the *Pfs47* genes in the GB4 and 7G8 lines that determine the survival of the GB4 in *A*. *gambiae* R strain. These amino acid differences (T236I, S242L, V247A and I248L) are localized in the second domain (D2) of Pfs47, between the two cysteines in that domain, that are 30 amino acids (aa) apart. In this manuscript, we analyzed the molecular basis of compatibility of *Pfs47* and *A*. *gambiae* by generating NF54 transformed parasites in which each of the four amino acid differences in *Pfs47* between the GB4 and 7G8 strains was introduced by replacing the residues in African NF54 parasites with those of the 7G8 line from Brazil, changing one amino acid at a time. Stable parasites that only differ by a single amino acid with the same genetic background were generated. We hypothesized that at least one of these single amino acid changes would reduce survival of NF54 parasites in *A*. *gambiae* R strain mosquitoes. The effect of these mutations on *A*. *albimanus* infection was also analyzed.

## Results

**Fig 1:**
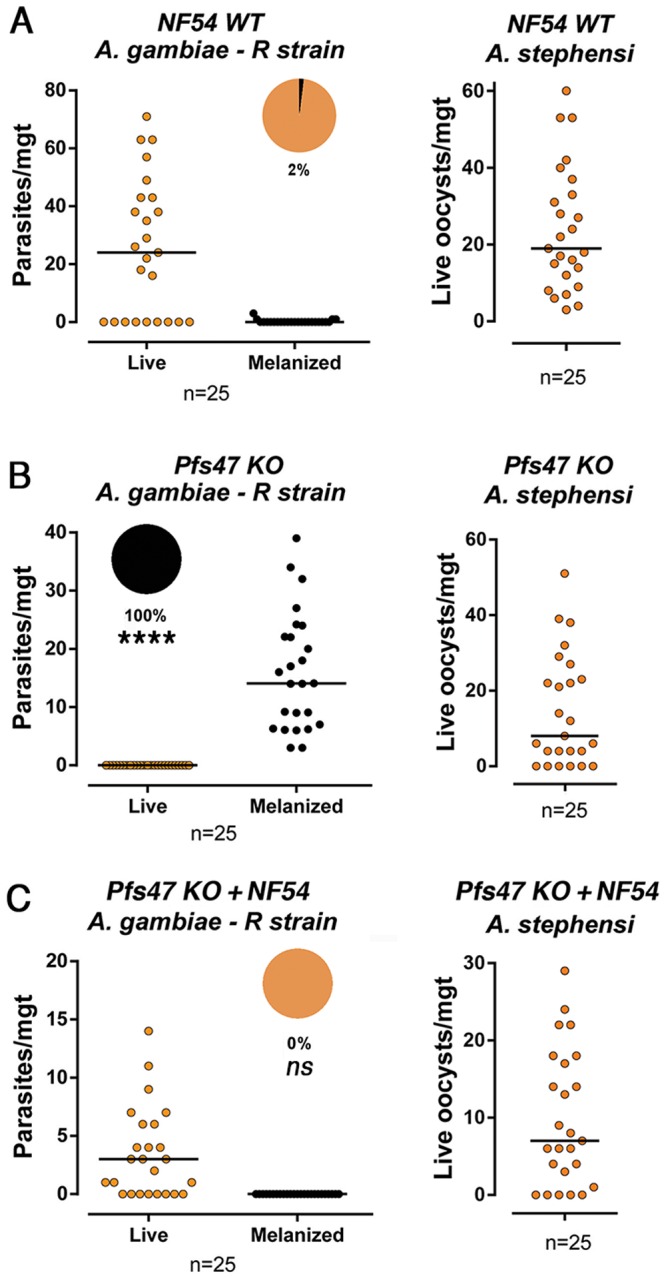
Infection phenotype of different *P*. *falciparum* lines (NF54 WT, Pfs47 KO, Pfs47 KO + NF54) in the *A*. *gambiae* R strain midgut 7–9 d postfeeding. Live and melanized parasites on individual mosquito midguts in the *A*. *gambiae* R strain and confirmation of gametocyte viability in the susceptible *A*. *stephensi* Nijmegen using *P*. *falciparum* NF54 (A), Pfs47 KO (B) and Pfs47 KO + NF54 (C). The medians are indicated with black lines and proportion of live (orange) and melanized (black) parasites are indicated with pie charts. Each dot represents the number of parasite on an individual mosquito and the median is indicated with a black line (n = number of midguts examined). The differences in the proportion of melanized parasites relative to NF54 WT were analyzed using the X^2^ test, **** p<0.0001, ns = not significant. All parasite phenotypes were confirmed in two independent experiments (S1 Fig).

We confirmed that most (98%) WT NF54 parasites survive in *A*. *gambiae* R strain mosquitoes (Fig 1A, S1A Fig). In each experiment the viability of the gametocyte culture was confirmed by infecting *A*. *stephensi* (Nijmegen Sda500) mosquitoes, a strain that has been genetically selected in the laboratory to be highly susceptible to *P*. *falciparum* (Welch) (Fig 1A–1C, S1A–S1C Fig) [12]. In contrast to NF54 WT parasites, a *P*. *falciparum Pfs47* KO NF54 line in which an *attB/attP* recombinase target site was included in the insertion that disrupts the gene [7] was completely (100%) melanized by *A*. *gambiae* R strain females (Fig 1B, S1B Fig). This is the same phenotype previously observed with an independent *Pfs47* KO NF54 line [11]. The *P*. *falciparum Pfs47* KO NF54 line was complemented with the NF54 *Pfs47* haplotype (Pfs47 KO+NF54) using the *attB/attP* recombinase target site to generate a stable parasite line. Genetic complementation with the *Pfs47* WT NF54 haplotype completely rescued parasite survival in the R strain (0% melanization) (Fig 1C, S1C Fig), confirming that the strategy was viable and that the stable transformants were functionally complemented.

**Fig 2:**
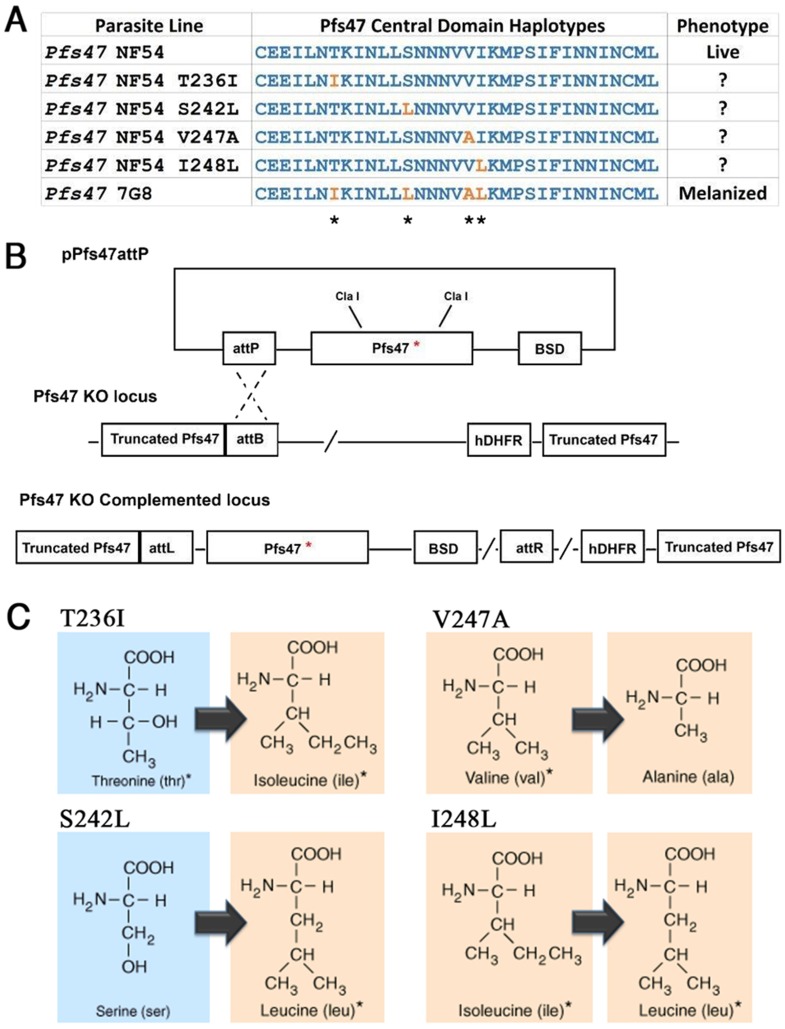
**(A) Schematic representation and alignment of Pfs47 Central Domain Haplotypes sequences used in this study.** Variable positions between the NF54 haplotype and the 7G8 haplotype along with the point mutations constructs used in this study to complement the Pfs47KO line are marked as orange. The known infection phenotype of these strains in the *A*. *gambiae* R strain midgut is shown on the right. (B) Schematic representation of the integrase-mediated complementation of different *Pfs47* haplotypes on the Pfs47KO locus by the plasmid pPfs47attP. The drug selection cassettes hDHFR and BSD the recombination adaptor sites attP/attB are showed. Representation is not in scale and is for illustration purposes only. (C) Schematic representation of the amino acids substituted on every single *Pfs47* point mutation construct. Nucleophilic amino acids are shaded in cyan and hydrophobic amino acids are shaded in orange.

The molecular basis of *Pfs47* phenotypic variation between the Africa and New World *P*. *falciparum* lines in the R strain was investigated by independently introducing each of the four single amino acid differences, one at a time, into the backbone of the previously reported p*Psf47*attP vector [7] using PCR-based mutagenesis (Fig 2A–2C). Following co-transfection of the *pPfs47*attP plasmids with an integrase-expressing plasmid and drug selection, the transgenic parasites complemented with the modified *Pfs47* constructs T236I, S242L, V247A and I248L were recovered and cloned by minimal dilution. Gene complementation was confirmed by PCR-amplification and sequencing; and gene copy number, quantitative mRNA and protein expression were also verified for all the lines (S3 Fig). We expected that the first two mutations, T236I and S242L would have a strong phenotypic effect, as they replace polar amino acids with hydrophobic ones that also have bulkier side chains (Fig 2C). The other two mutations (V247A and I248L) are more conservative, as they do not alter the polarity of the side chains; and in the case of I248L the only difference is the position of a methyl group in the side chain (Fig 2C).

**Fig 3:**
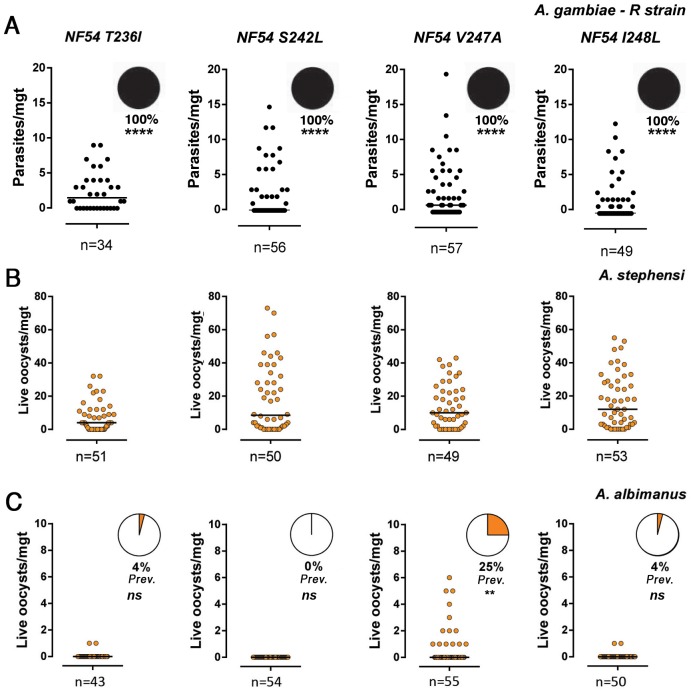
Infection phenotype of different *Pfs47* complement *P*. *falciparum* lines (T236I, S242L, V247A and I248L) in the *A*. *gambiae* R strain, *A*. *albimanus* and *A*. *stephensi* 7–9 d post-feeding representing two pooled independent experiments (Experiment 1 and Experiment 2, S1–S3 Tables). (A) Infection of *A*. *gambiae* R strain. The pie charts indicate the proportion of live (orange) and melanized (black) parasites. (B) Infection of *A*. *stephensi* Nijmegen, and (C) *A*. *albimanus* mosquitoes with *P*. *falciparum* NF54 Pfs47KO complemented derivatives expressing *Pfs47* haplotypes NF54 T236I, S242L, V247A and I248L. The orange area of the pie charts indicates the prevalence of infection in *A*. *albimanus*. Each dot represents the number of parasite on an individual mosquito and the median is indicated with a black line (n = number of midguts examined). Melanization prevalences in *A*. *gambiae* R strain were compared with the X^2^ test relative to Pfs47 KO + NF54, infection prevalences in *A*. *albimanus* were compared with the X^2^ test, ns = no significant difference,** p<0.01, **** p<0.0001. All parasite phenotypes were confirmed in two or three independent experiments.

As expected, the two lines in which *Pfs47* was mutated with a non-conservative single amino acid replacement (T236I and S242L) were no longer functionally complemented, and were unable to infect the *A*. *gambiae* R strain (100% melanized) (Fig 3A, S1 Table). We were surprised that the other two lines with more conservartive mutations (V247A and I248L) had a similar phenotype. The quality of the culture and the viability of the complemented lines was confirmed by infecting *A*. *stephensi* Sda500 mosquitoes side-by-side with the same cultures (Fig 3B, S3 Table). These results indicated that there is a strong specificity between the African Pfs47 protein of the parasite and the putative mosquito receptor, as any amino acid substitution, even a conservative one, disrupts the ability of WT NF54 *Pfs47* to evade the immune system of the *A*. *gambiae* R strain.

**Fig 4:**
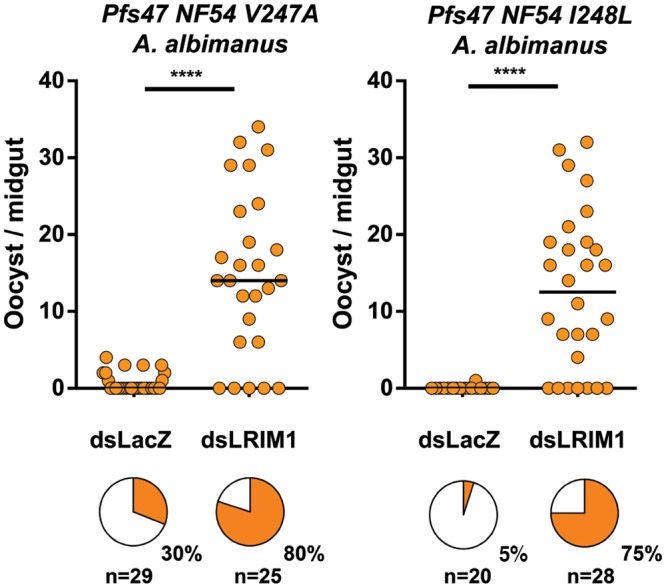
Effect of LRIM1 silencing in *A*. *albimanus* mosquitoes on its infection with *P*. *falciparum Pfs47* NF54 V247A and I248L. Effect of silencing the leucine-rich repeat immune protein 1 (LRIM1) in *A*. *albimanus* on infection with NF54 *Pfs47* haplotypes V247A and I248L. Each dot represents the number of parasite on an individual mosquito and the median is indicated with a black line (n = number of midguts examined). The orange area of the pie charts indicates the prevalence of infection. All parasite phenotypes were confirmed in two independent experiments. Median number of oocysts were compared using the Mann–Whitney test. ****P < 0.0001.

We have previously shown that the *Pfs47* KO line cannot infect *A*. *albimanus* mosquitoes (0% prevalence of infection), but introducing the American-7G8 *Pfs47* haplotype (that differs from the African-GB4 line only in these four amino acids) greatly enhances infection reaching a prevalence of 63% in this New World vector. We tested whether introducing one amino acid change at a time could enhance infection in *A*. *albimanus*. In this mosquito the complement system lyses the parasites and no melanizations are observed. The S242L mutation had no effect (0% prevalence) (Fig 3C, S2 Table), the T236I and I248L substitutions had a very minor effect (2–4% prevalence, not significant, χ^2^ test), while the V247A mutation had a stronger effect enhancing infection and reaching a prevalence of infection of 25–30% (p<0.01, χ^2^ test, Figs 3C and 4, S2 Fig, S2 Table). We then tested whether the *A*. *albimanus* complement-like system was activated and limiting infection with two partially rescued lines. Disruption of the complement system by silencing LRIM1 greatly enhanced the intensity of infection with both lines (p< 0.0001, Fig 4, S2 Fig). The effect on the I248L lines was stronger, as the prevalence of infection increased from 5% in the LacZ control to 75% (37.5 fold) in the LRIM1-silenced group, while in the V247A line the prevalence increased from 30% to 80% (2.6 fold)(Fig 4, S2 Fig).

## Discussion

Global analysis of 364 *P*. *falciparum* isolates identified 42 different *Pfs47* haplotypes with strong geographic structure and differences of compatibility between isolates from Africa, Asia, or the Americas and the mosquito vectors from these regions [7]. In general, parasites were more effective infecting mosquitoes from the same continent they originated from, suggesting that mosquito vectors select the parasites genotypes that circulate in a given geographic area. Furthermore, we demonstrated that incompatible parasites are eliminated by the mosquito complement-like system and that replacing the *Pfs47* haplotype expressed in a parasite was sufficient to change the compatibility with a given vector. Based on these observations the “Lock and Key” model of *Plasmodium* mosquito immune evasion was proposed stating that: “We can think of *Pfs47* as a “key” that allows the parasite to “turn off” the mosquito detection system by interacting with some mosquito receptor protein(s) (the lock). There are different haplotypes of this key, and the parasite needs to have the right key for the “lock” present in a given mosquito species to survive, continue to be transmitted, and become established in a new region” [7].

In this manuscript we explored how precise the fit of the *Pfs47* “key” with the mosquito receptor has to be for the parasite to evade immunity. Our studies indicate that, in the *A*. *gambiae* R strain, only a perfect fit of four critical amino acids in the central domain would do, as any amino acid modification, even two different conservative single amino acid replacements completely disrupted the ability of the parasites to hide from the mosquito immune system and they were all destroyed. The V247A mutation is very interesting, because it greatly reduced parasite survival in the *A*. *gambiae* R strain, but had the opposite effect in *A*. *albimanus* and enhanced infection. This indicates that this is a critical amino acid and that the two additional methyl groups in the side chain of the valine residue were probably preventing an effective interaction with the putative Pfs47 receptor in *A*. *albimanus*. However, the effect of this modification on immune evasion was partial, as disrupting the mosquito complement-like system, by silencing LRIM1 in *A*. *albimanus*, greatly enhanced the level of infection, indicating that many parasites are still detected and destroyed by the mosquito immune system. It should be noted that when a NF54 KO line is complemented with the *Pfs47* haplotype with the four amino acids present in the New World 7G8 line, the parasite becomes “invisible” to the mosquito and disrupting the complement system has no effect on the infection [7]. Furthermore, all the *Pfs47* haplotypes identified so far in the New World share the four critical amino acids present in the 7G8 haplotype [7]. Taken together, our findings indicate that besides the V247A mutation, the other three amino acids are also required for complete immune evasion in *A*. *albimanus*.

We have previously shown that the JNK pathway is a major effector of mosquito antiplasmodial immunity, as it regulates the expression of the enzymes that mediate midgut epithelial nitration in response to *Plasmodium* invasion [13]; and epithelial nitration is necessary for effective activation of the mosquito complement-like system [14]. The R strain is in a chronic state of oxidative stress that is enhanced following a blood meal [15], and exhibits a constitutive level of systemic activation of JNK signaling with increased expression of the enzymes that mediate midgut nitration (NOX5 and HPX2) and of the antiplasmodial effectors TEP1 and FBN9 in hemocytes [13]. Furthermore, disruption of JNK signaling in the R strain greatly enhances infection with *P*. *berghei*, increasing the prevalence of infection from 0 to 68% [13].

A detailed functional analysis of the mosquito immune response to WT and *Pfs47* KO *P*. *falciparum* (NF54 genetic background) revealed that *Pfs47* allows the parasite to evade immunity by disrupting JNK signaling and preventing activation of epithelial nitration in response to ookinete midgut invasion [16]. The higher basal level of JNK activation in the R strain seems to pose a greater challenge to the parasite and may explain the observed results. A high affinity interaction with the mosquito *Pfs47* receptor that triggers the disruption of JNK signaling appears to be required for *P*. *falciparum* to survive in mosquitoes with the R strain genetic background. Even minor modifications, such as the V247A or the I248L mutations, that could reduce the binding affinity, but are not predicted to have major effects on the overall molecular interaction, disrupt the parasite’s ability to block the mosquito antiplasmodial response. This level of specificity supports that idea that, in *A*. *gambiae* R strain, *Pfs47* functions like a “key” that must fit the receptor and suggests that the region between the two cysteines in the D2 domain, where all the amino acid differences were present, is crucial for the interaction with the putative receptor in the mosquito midgut. A recent report identified an African *P*. *falciparum* line (NF165) in which the sequence of the D2 domain of *Pfs47* is identical to that of NF54 and GB4 parasites, but most parasites (up to 66%) are melanized in the R strain [17]. Unfortunately, only 59% of the NF165 *Pfs47* gene sequence was determined [17], and a recent global analysis of *P*. *falciparum* lines identified 11 non-synonymous polymorphisms in the region that was not sequenced [7]. It is thus possible that some polymorphism(s) are present in this region of the NF165 line (but were not sequenced) and this may be sufficient to disrupt the interaction with the receptor. Alternatively, a compatible *Pfs47* haplotype may be necessary, but not sufficient for parasites to survive. Polymorphisms in other parasite genes present in the lines analyized by Eldering *et al*. [17] may also be critical for *Plasmodium* survival. The parasite may also require, for example, a higher capacity to detoxify reactive oxygen species (ROS), as the JNK-mediated induction of NOX5 expression generates high levels of intracellular ROS as the parasites traverse the mosquito midgut epithelial cell.

Some vectors seem to be more permissive, for example, the *A*. *gambiae* G3 strain can be infected with both NF54 and 7G8 parasites. However, it is more susceptible to NF54 infection than to 7G8 [7]. Conversely, *A*. *albimanus* is more susceptible to 7G8 than to NF54 [7]. The *A*. *stephensi* (Nijmegen Sda500) strain, that was genetically selected in the laboratory to be highly susceptible to *P*. *falciparum* (Welch), is highly susceptible to infection with all Pfs47 haplotypes tested so far, and is readily infected even with Pfs47 NF54 KO parasites (Fig 1B, S1B Fig). At the other end of the spectrum, in *A*. *gambiae* R strain, there is no room for variation in the four critical amino acids in the *Pfs47* central domain and it may represent an extreme case in which only a perfect fit of *Pfs47* with its receptor allows *P*. *falciparum* to survive. The goal of this study was to minimize as much as possible the genetic variation, both in the vector and parasite, that could influence the phenotypes, so that one could asses the effect of single amino acid differences on vector/parasite compatibility and transmission. The observed results with the anopheline laboratory lines used in this study cannot be generalized to all mosquitoes of a given species. However, the observation that the NF54 V247A mutation disrupts the ability of the parasite to evade immunity in the *A*. *gambiae* R strain and results in complete elimination, but has the opposite effect on *A*. *albimanus*, enhancing infection, supports the hypothesis that the “correct key” (*Pfs47* haplotype) that the parasite needs is different between evolutionary distant anopheline vectors of malaria. Understanding these complex vector/parasite interactions, under natural field conditions, would require direct genetic analysis of the *P*. *falciparum Pfs47* haplotypes present on individual naturally-infected anopheline mosquitoes and, ideally, simultaneous analysis of the mosquito *Pfs47* receptor variant in the same sample. We conclude that malaria transmission involves a complex interplay between the genetic background of the parasite and the mosquito and that *Pfs47* can be critical in this interaction, as it mediates *Plasmodium* immune evasion through molecular interactions that need to be very precise in some parasite/vector combinations.

## Materials and Methods

### *Anopheles* Mosquitoes and *Plasmodium* Parasites

*Anopheles gambiae* L35 (R strain), *Anopheles albimanus* and *Anopheles stephensi* Nijmegen Sda500 strains were used. Mosquitoes were reared at 27°C and 80% humidity on a 12-h light-dark cycle under standard laboratory conditions. The *Plasmodium falciparum* strains used—NF54, NF54-Pfs47KO and Pfs47 complemented lines (Africa-NF54, NF54 T236I, NF54 S242L, NF54 V247A and NF54 I248L)—were maintained in O+ human erythrocytes using RPMI 1640 medium supplemented with 25 mM HEPES, 50 mg/l hypoxanthine, 25 mM NaHCO3, and 10% (v/v) heat-inactivated type O+ human serum (Interstate Blood Bank, Inc., Memphis, TN) at 37°C and with a gas mixture of 5% O2, 5% CO2, and balance N_2_ [11].

### Experimental Infection of Mosquitoes with *P*. *falciparum*

Mosquito females were infected artificially by membrane feeding with *P*. *falciparum* gametocyte cultures. Gametocytogenesis was induced as previously described [11]. Mature gametocyte cultures (stages IV and V) that were 14–16 d were used to feed mosquitoes using membrane feeders at 37°C for 30 min. Midguts were dissected 8 d after feeding, and oocysts were stained with 0.1% (wt/vol) mercurochrome in water and counted by light microscopy. Median numbers of oocysts were compared using the Mann-Whitney test; infection prevalences and the proportion of melanized parasties were compared with the X^2^ test. All parasite phenotypes were confirmed in two to three independent experiments.

### dsRNA-Mediated Gene Knockdown

Individual female *A*. *albimanus* mosquitoes were injected 1–2 d postemergence as previously described [7]. Briefly, mosquitoes were injected with 69 nL 3 μg/μL dsRNA solution 3–4 d before receiving a *Plasmodium*-infected blood meal. dsRNA *A*. *albimanus* LRIM1 were produced using the MEGAscript RNAi Kit (Ambion, Austin, TX) using DNA templates obtained by PCR using *A*. *albimanus* cDNA and the primers previously described [7] with T7 polymerase promoter sites added in the 5ˊ-end. *A*. *albimanus* LRIM1 gene silencing was assessed in whole sugar-fed mosquitoes by quantitative real-time PCR using primers AaLRIM1-qF, AaLRIM1-q, respectively and were found to be 80% lower in dsRNA immune genes injected mosquitoes compared with a dsLacZ-injected control.

### Stable Genetic Complementation of *P*. *falciparum Pfs47KOattB*

The generated Pfs47KOattB line was complemented with different point mutated alleles of NF54-Pfs47 with the plasmid pPfs47attP [7]. Due to the inability of generating point mutations in the 8.3 kb pPfs47attP, the domain two of the *Pfs47* from *P*. *falciparum* NF54 was PCR amplified with the In-Fusion^®^ designed oligonucleotides prClaF and prClaR and cloned into a pGemT-easy plasmid, generating pGemT-easy-Pfs47D2 plasmid. Point mutations were generated in the latter plasmid using the QuickChange Point Mutagenesis kit (Agilent) with the primers T236I_sense and T236I_antisense; S242L_sense and S242L_antisense, V247A_sense and V247A_antisense; I248L-sense and I248L_antisense, respectively. Then the point mutated fragment was PCR amplified with the primers prClaF and prClaR and introduced by infusion into the ClaI digested pPfs47attP plasmid to generating pPfs47attP NF54 T236I, NF54 S242L, NF54 V247A and NF54 I248L. Cloning procedures were carried out in TOP10F bacteria (Invitrogen) and every construct was fully sequenced (Operon).

*P*. *falciparum Pfs47KO* was cotransfected with a particular pPfs47attP (NF54 T236I, NF54 S242L, NF54 V247A or NF54 I248L) and the pINT—which encodes for the mycobacteriophage Bxb1 serine integrase in order to catalyze the integration of the plasmid pPfs47attP [18]—as previously describe with modifications [19]. Briefly, transfection mixture was prepared at room temperature and consisted of 20 μl packed RBCs, 5 μg of each plasmid DNA (1μg/ul) (Qiagen plasmid Maxiprep kit) and of 70 μl Amaxa SE solution resulting in a final volume of 100 μl. The transfection mixture was transferred to an Amaxa Nucleocuvette (Lonza) and transfection was performed at room temperature using an Amaxa 4D-Nucleofector. After applying the CM-162 pulse, electroporated RBC’s were processed as mentioned above. Culture media was changed daily and selection drugs (2.5 μg/ml Blasticidin HCl and 200μg/ml Geneticin) were added 24 hours after electroporation and maintained continuously in the asexual cultures unless stated otherwise. Once parasitemia was observed (day 21–28), parasites were cloned by minimal dilution in 96 wells plates (day 1). On day 14, 5ul aliquots of the 96 well plates were incubated with 2X SYBR-green-1 (Invitrogen) and 165 nM MitoTracker Deep Red (Invitrogen) in PBS and measure of live parasite were assessed using flow cytometry [7]. Positive wells were transferred to 24 well plates and later to 6 well plate.

### Nucleic Acid Analysis

Primers pairs prIA1’F2/prIA1’R and prIA2’F/prIA2’R were used to detect both integration arms 1 and 2 respectively of the *pPfs47attP’*s plasmids into the Pfs47KO locus on chromosome 13. *Pfs47* gene copy number was estimated by testing each DNA sample by qPCR with the primers PF13_0248F and PF13_0248R in parallel against the *P*. *falciparum* gene Pf10_0203 (ADP-ribosylation factor) gene using the primers 0203F and 0203R [7]. Pfs47 mRNA expression in the lines used in this work was confirmed by qPCR on cDNA from stage IV-V gametocyte cultures using the mentioned primers as previously described [7]. Estimation of gene dosage and mRNA expression was calculated according to the ^ΔΔ^Ct method [20].

## Supporting Information

S1 Fig Replicates of infection phenotype of different *P*. *falciparum* lines (NF54 WT, Pfs47 KO, Pfs47 KO + NF54) in the *A*. *gambiae* R strain and *A*. *stephensi*. (DOCX)

S2 Fig Replicate of the effect of LRIM1 silencing in *A*. *albimanus* mosquitoes on its infection with *P*. *falciparum Pfs47* NF54 V247A and I248L. (DOCX)

S3 Fig Nucleic acid and protein analysis of the *P*. *falciparum* NF54 Pfs47KO complemented derivatives Pfs47 NF54 T236I, S242L, V247A and I248L. (DOCX)

S1 Table Independent experiments of infection phenotypes of different *Pfs47* complement *P*. *falciparum* lines (PfsKO+NF54, T236I, S236L, V247A and I248L) in *A*. *gambiae* R strain. (DOCX)

S2 Table Independent experiments of infection phenotypes of different *Pfs47* complement *P*. *falciparum* lines (PfsKO+NF54, T236I, S236L, V247A and I248L) in *A*. *albimanus*. (DOCX)

S3 Table Independent experiments of infection phenotypes of different *Pfs47* complement *P*. *falciparum* lines (PfsKO+NF54, T236I, S236L, V247A and I248L) in *A*. *stephensi*. (DOCX)

S4 Table Sequence of primers used in this study. (DOCX)

## Abbreviations

APL1: *Anopheles Plasmodium*-responsive leucine-rich repeat 1 protein
JNK: Janus N-terminal Kinase
LRIM1: leucine-rich repeat immune protein 1
TEP1: Thioester-containing Protein
