# Supplementary material for: Molecular Analysis of Pfs47-Mediated Plasmodium Evasion of Mosquito Immunity
Source: PLoS One. 2016 Dec 19;11(12):e0168279. doi: 10.1371/journal.pone.0168279 (PMC5167319; doi:10.1371/journal.pone.0168279)
Supplement: S1 Fig — (DOCX) [file pone.0168279.s001.docx]

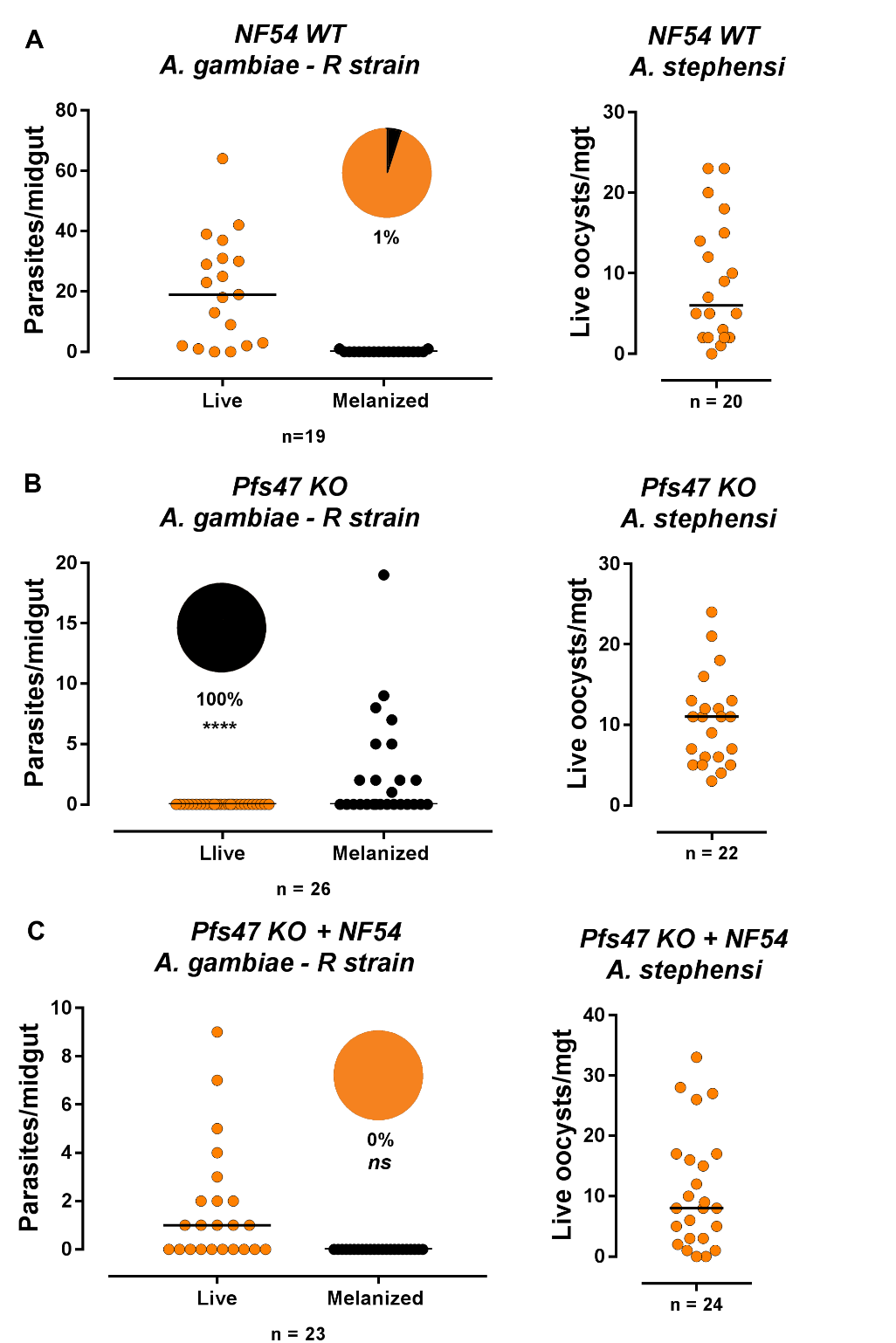


S1 Fig. Infection phenotype of different *P. falciparum* lines (NF54 WT, Pfs47 KO, Pfs47 KO + NF54) in the *A. gambiae* R strain midgut 7–9 d postfeeding. Live and melanized parasites on individual mosquito midguts in the *A. gambiae* R strain and confirmation of gametocyte viability in the susceptible *A. stephensi* Nijmegen using *P. falciparum* NF54 (A), Pfs47 KO (B) and Pfs47 KO + NF54 (C). The medians are indicated with black lines and proportion of live (orange) and melanized (black) parasites are indicated with pie charts. Each dot represents the number of parasite on an individual mosquito and the median is indicated with a black line (n = number of midguts examined). The differences in the proportion of melanized parasites relative to NF54 WT were analyzed using the χ^2^ test, **** p<0.0001, ns= not significant. All parasite phenotypes were confirmed in two independent experiments.
