# Supplementary material for: Molecular Analysis of Pfs47-Mediated Plasmodium Evasion of Mosquito Immunity
Source: PLoS One. 2016 Dec 19;11(12):e0168279. doi: 10.1371/journal.pone.0168279 (PMC5167319; doi:10.1371/journal.pone.0168279)
Supplement: S2 Fig — (DOCX) [file pone.0168279.s002.docx]

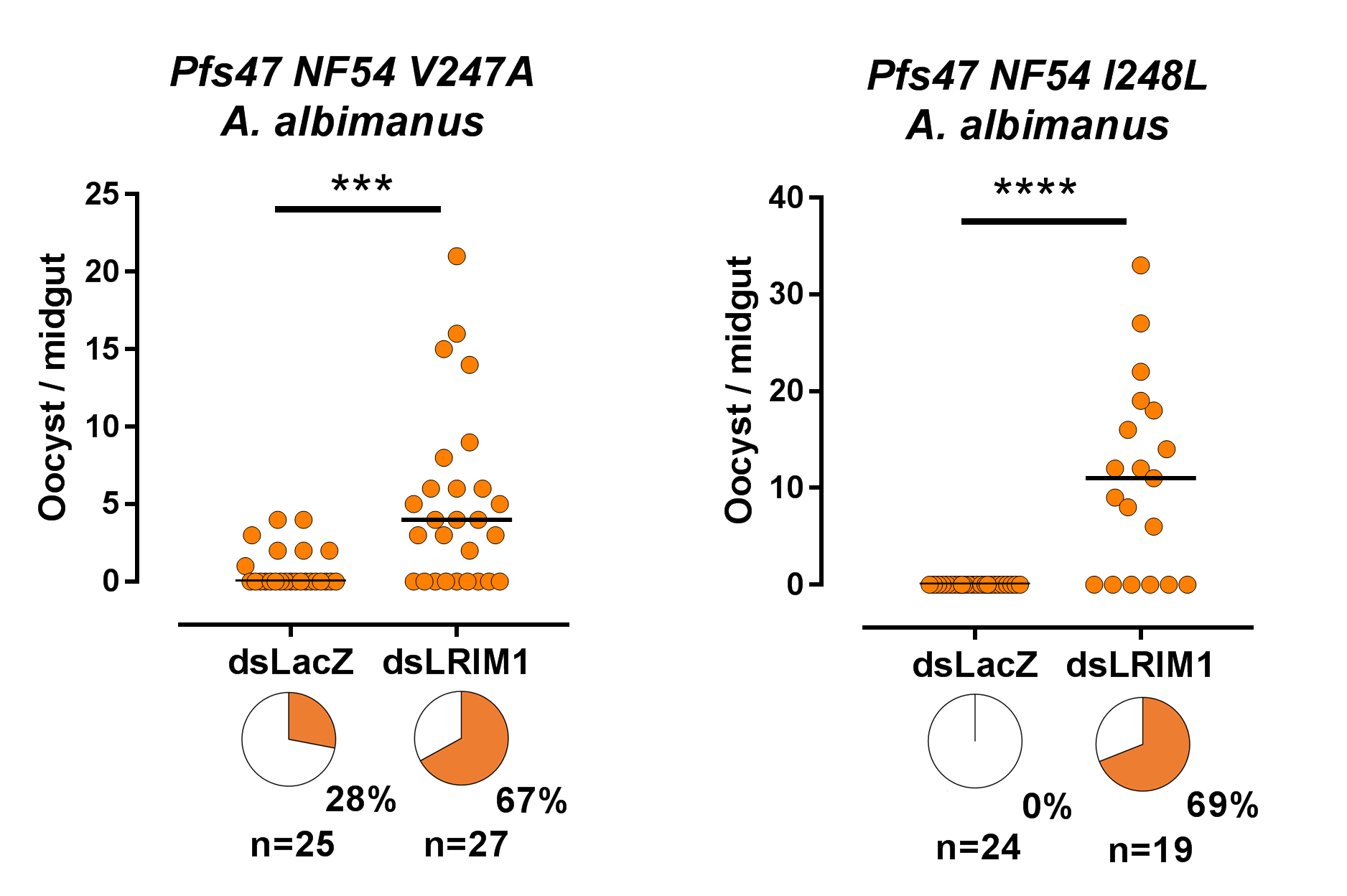


S2 Fig. Effect of LRIM1 silencing in *A. albimanus* mosquitoes on its infection with *P. falciparum* Pfs47 NF54 V247A and I248L. Effect of silencing the leucine-rich repeat immune protein 1 (LRIM1) in *A. albimanus* on infection with NF54 Pfs47 haplotypes V247A and I248L. Each dot represents the number of parasite on an individual mosquito and the median is indicated with a black line (n = number of midguts examined). The orange area of the pie charts indicates the prevalence of infection. Medians were compared using the Mann–Whitney test. ***P<0.001, ****P < 0.0001.
