# Supplementary material for: Molecular Analysis of Pfs47-Mediated Plasmodium Evasion of Mosquito Immunity
Source: PLoS One. 2016 Dec 19;11(12):e0168279. doi: 10.1371/journal.pone.0168279 (PMC5167319; doi:10.1371/journal.pone.0168279)
Supplement: S3 Fig — (DOCX) [file pone.0168279.s003.docx]

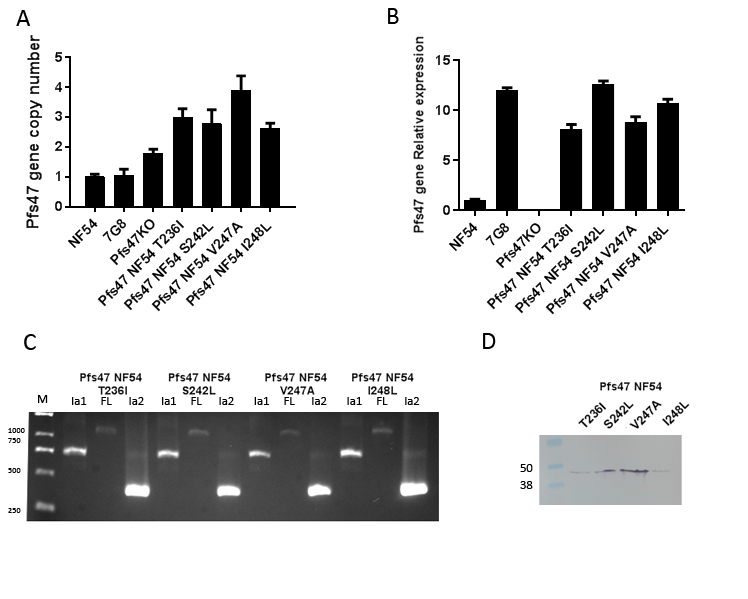


S3 Fig. Nucleic acid and protein analysis of the *P. falciparum* NF54 Pfs47KO complemented derivatives Pfs47 NF54 T236I, S236L, V247A and I248L. (A) Pfs47 gene copy number of the different lines generated was assessed by qPCR. Primers were designed to amplify a 84bp sequence included in the truncated Pfs47 KO in order to detect one copy in the wild type strain, two in the Pfs47KO and three in the complemented derivatives. (B) Relative mRNA expression of Pfs47 in wild-type (7G8 and NF54), Pfs47 KO, and the complemented derivatives Pfs47 NF54 T236I, S236L, V247A and I248L. Relative mRNA expression of Pfs47 was assessed by qPCR in stage IV–V gametocyte cultures. (C) Genotype confirmation of the complementation of the Pfs47KO. Integration-specific PCR products after cloning of parasites by minimal dilution, based on different combination of the primers pairs to detect Integration arm 1 (Ia1), full length Pfs47 (FL) and Integration arm 2 (Ia2) using extracted gDNA from the complemented derivatives Pfs47 NF54 T236I, S236L, V247A and I248L. (D) Western blot analysis of expression of Pfs47 protein in equivalent amounts of gametocyte cultures from the complemented derivatives Pfs47 NF54 T236I, S236L, V247A and I248L. Detection of Pfs47 protein in the complemented lines confirms gene expression upon complementation of the Pfs47KO line.
