# Supplementary material for: Molecular Analysis of Pfs47-Mediated Plasmodium Evasion of Mosquito Immunity
Source: PLoS One. 2016 Dec 19;11(12):e0168279. doi: 10.1371/journal.pone.0168279 (PMC5167319; doi:10.1371/journal.pone.0168279)
Supplement: S2 Table — (DOCX) [file pone.0168279.s005.docx]

S2 Table. Infection phenotype of different Pfs47 complement *P. falciparum* lines (T236I, S236L, V247A and I248L) in *A. albimanus* 7–9 d post-feeding. Table shows prevalence of infection (%). Infection prevalences in *A. albimanus* were compared with the χ^2^ test, ns= no significant difference, ** p<0.01. Pooled results from Experiment 1 and 2 are also presented in Figure 3 in the main text.
